# The Influence of Cardiac Arrest Floor-Level Location within a Building on Survival Outcomes

**DOI:** 10.3390/jpm13081265

**Published:** 2023-08-16

**Authors:** Chiwon Ahn, Young Taeck Oh, Yeonkyung Park, Jae Hwan Kim, Sojune Hwang, Moonho Won

**Affiliations:** 1 Department of Emergency Medicine, College of Medicine, Chung-Ang University, Seoul 06974, Republic of Korea; cahn@cau.ac.kr (C.A.); thesult@cauhs.or.kr (J.H.K.); junee327@naver.com (S.H.); wonmh0922@cauhs.or.kr (M.W.); 2 Department of Emergency Medicine, Hallym University Dongtan Sacred Heart Hospital, Hwaseong 18450, Republic of Korea; powerfreeze@hanmail.net; 3 Division of Pulmonary and Critical Care Medicine, Department of Internal Medicine, Veterans Health Service Medical Center, Seoul 05368, Republic of Korea; 4 Department of Internal Medicine, College of Medicine, Hanyang University, Seoul 04763, Republic of Korea

**Keywords:** building floor, emergency medical services, out-of-hospital cardiac arrest, survival, vertical location

## Abstract

This nationwide, population-based observational study investigated the association between the floor level of out-of-hospital cardiac arrest (OHCA) incidence and survival outcomes in South Korea, notable for its significant high-rise apartment living. Data were collected retrospectively from OHCA patients through the South Korean Out-of-Hospital Cardiac Arrest Surveillance database. The study incorporated cases that included the OHCA’s building floor information. The primary outcome assessed was survival to discharge, analyzed using multivariate logistic regression, and the secondary outcome was favorable neurological outcome. Among 36,977 patients, a total of 29,729 patients were included, and 1680 patients were survivors. A weak yet significant correlation between floor level and hospital arrival time was observed. Interestingly, elevated survival rates were noted among patients from higher floors despite extended emergency medical service response times. Multivariate analysis identified age, witnessed OHCA, shockable rhythm, and prehospital return of spontaneous circulation (ROSC) as primary determinants of survival to discharge. The floor level’s impact on survival was less substantial than anticipated, suggesting residential emergency response enhancements should prioritize witness interventions, shockable rhythm management, and prehospital ROSC rates. The study underscores the importance of bespoke emergency response strategies in high-rise buildings, particularly in urban areas, and the potential of digital technologies to optimize response times and survival outcomes.

## 1. Introduction

Out-of-hospital cardiac arrest (OHCA) is a medical emergency that necessitates swift intervention for optimal patient outcomes [1,2,3,4]. In addition, previous studies have established a correlation between survival rates following OHCA and the duration of emergency medical service (EMS) access to patients. This access time is influenced by various factors, including the EMS system, density of ambulances, and the location of the cardiac arrest incident [5,6,7]. In South Korea, a country characterized by a significant urban population residing in high-rise apartment buildings, it is crucial to understand the characteristics of OHCA in association with housing type. The prevalence of apartment living in these urban regions profoundly impacts the OHCA characteristics within the context of various South Korean housing types. These circumstances could potentially present unique challenges for EMS in their response to OHCA incidents. More specifically, the vertical location of an incident within a high-rise building could influence response times and, consequently, patient outcomes [8,9,10,11,12,13].

Previous studies have explored the vertical location of OHCA occurrence and its association with clinical outcomes [8,9,12]. Research from various regions has scrutinized variables like response time, survival rates, and the prevalence of bystander interventions. In Japan, a previous study reported that neurologically favorable outcomes one month after OHCA were less frequent among individuals on higher floors (third floor or more) compared to those on lower floors (below third floor) [8]. In Singapore, other study showed that the floor level where a cardiac arrest occurs is linked to survival probability, with basements, ground floors, and extreme upper floors showing the highest survival rates, while midrange floors show lower survival rates [9]. The findings from these studies suggest that factors related to the vertical location of OHCA, bystander intervention, and EMS response time significantly affect the success of resuscitation efforts and the subsequent clinical outcomes [8,9,13].

Considering the unique housing landscape in South Korea and the potential influence of vertical location on OHCA outcomes, further research is imperative. This study aims to investigate the relationship between the floor level of an OHCA occurrence and survival rates within high-rise residential areas in South Korea. Previous research has indicated disparities in emergency medical services (EMS) response times depending on the floor level [8,9,10,11,12,13], yet the specific impact these variations have on survival rates remains unclear. The purpose of this research is to elucidate this mechanism and provide information necessary for improving EMS response strategies and building safety protocols within high-rise buildings. The study also seeks to explore other survival predictors in the vertical location setting. Through this investigation, we aim to contribute to the enhancement of emergency medical response strategies in high-rise buildings and the increase in survival rates among OHCA patients.

## 2. Materials and Methods

### 2.1. Study Design, Setting, and Data Source

This retrospective observational study used the nationwide, population-based Out-of-Hospital Cardiac Arrest Surveillance database, managed by the Korea Disease Control and Prevention Agency (https://www.kdca.go.kr/ accessed on 30 July 2023), to evaluate the impact of OHCA vertical location on survival rates. The database contains information on all acute cardiac arrest patients transported to medical facilities by EMS, amounting to approximately 30,000 patients annually from 2016 through 2021. As the study data were anonymized, the Korea Disease Control and Prevention Agency approved the research use of this database, and the relevant institutional review board exempted this work from assessment.

In South Korea, the government-operated public EMS, administered by 19 fire headquarters under the National Fire Agency, is available 24/7 [14]. The EMS that responds to the scene consists of emergency medical technicians (EMT), occasionally an emergency nurse practitioner, and, for advanced medical guidance, a physician of emergency medicine [15]. An ambulance is dispatched to the OHCA location upon receipt of a call, and the patient is subsequently transported to the hospital. Before hospital arrival, EMTs administer cardiopulmonary resuscitation (CPR) using an automated external defibrillator (AED). Under a physician’s supervision, CPR can be halted, or advanced airway techniques can be applied, but drugs for advanced life support cannot be administered. EMTs relay all pertinent information to the hospital during patient transfer. Resuscitation treatments at the hospital and following the return of spontaneous circulation (ROSC) are administered per each hospital’s protocol.

The Out-of-Hospital Cardiac Arrest Surveillance database incorporates patient data drawn from the EMS data registry and hospital medical records. Medical record investigators from the Korea Disease Control and Prevention Agency visit medical facilities to scrutinize arrest patients’ medical records concerning treatments and outcomes, as well as to verify compliance with the Utstein Style [16] and the Resuscitation Outcomes Consortium Project [17]. Using a custom survey form, the database records individual and setting data, as well as data on EMS, emergency department care, hospital procedures, and discharge outcomes, including survival and neurological outcomes.

### 2.2. Study Population and Classification of Arrest Location

We included OHCAs with known building floor occurrence information. Patients with do-not-resuscitate orders, traumatic cardiac arrests, invalid prehospital data, and those transferred to other facilities were excluded. Building floor information ranged from 9 floors below ground to 55 floors above ground. For efficient analysis, we consolidated basement floors into one category and grouped together floors from the 16th floor and above.

### 2.3. Variables

Collected variables included age, sex, witness status of the arrest, whether bystander CPR and/or AED was administered, initial rhythms during the prehospital interval (non-shockable vs. shockable), and prehospital and in-hospital ROSC. A shockable rhythm was defined as an initial rhythm that was either pulseless ventricular tachycardia or ventricular fibrillation. We examined variables related to OHCA location, such as place (residential or public), urbanity, and building floors. Prehospital variables included hospital arrival time and dispatch assistance. Hospital arrival time was defined as the interval from the call to hospital arrival. Additionally, information on underlying diseases, and treatments including percutaneous coronary intervention, target temperature management, pacemaker, and extracorporeal membrane oxygenation, and outcomes in the hospital was collected.

### 2.4. Outcome Measures

The primary outcome of this study was survival to discharge, defined as a patient’s regular discharge or transfer to another healthcare facility for ongoing care following acute treatment. Secondary outcomes were neurologic outcome and hospital arrival time. Neurologic results were classified using the Cerebral Performance Category (CPC) score, with CPC scores of 1 and 2 considered indicative of favorable neurologic outcomes.

### 2.5. Statistical Analyses

Data were analyzed using Excel 2019 (Microsoft, Redmond, WA, USA) and R version 4.3.1 (R Foundation for Statistical Computing, Vienna, Austria). Descriptive statistics were applied to describe the baseline characteristics. Continuous variables are expressed as means ± standard deviations or median with interquartile range. Student’s *t*-test was used to analyze normally distributed variables (between group comparisons). Categorical variables are expressed as frequencies and percentages, and the χ^2^ test or Fisher’s exact test was used to analyze these using contingency tables. To identify outcome predictors, covariates were evaluated via multivariate analysis. Logistic regression using the “enter” method was independently performed. Sex, age, urbanity, building floor, witnessed OHCA, bystander CPR, shockable rhythm, cardiogenic cardiac arrest, public place, prehospital ROSC, and hospital arrival time were adjusted for. A *p* value < 0.05 was considered statistically significant.

## 3. Results

**Figure 1:**
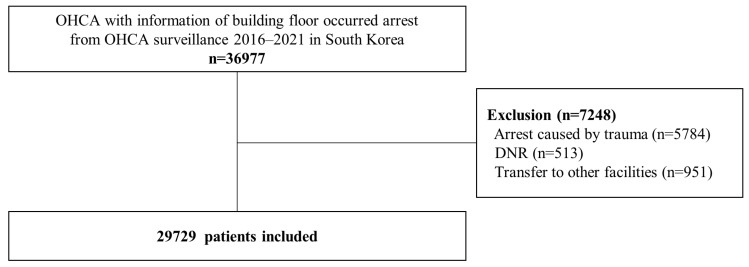
Flow diagram of study population enrollment. DNR, do-not-resuscitate; OHCA, out-of-hospital cardiac arrest.

From January 2016 through December 2021, we identified 36,977 patients with available data on the building floor of arrest occurrence. After excluding patients with do-not-resuscitate orders (n = 513), those with trauma-related cardiac arrests (n = 5784), and those transferred to other facilities (n = 951), we included 29,729 patients in our study, following the exclusion of 7248 patients (Figure 1).

The survivors and non-survivors exhibited significant differences from one another. The mean age of survivors was 55.4 ± 17.0, while that of non-survivors was 68.9 ± 18.3. Urban regions were the places of residence of 98.3% of the deaths and 99.3% of the survivors (*p* = 0.004). Compared with non-survivors, survivors had significantly higher rates of witnessed cardiac arrests, shockable rhythms, and bystander CPR. Additionally, a prehospital ROSC was observed in 2.0% of the deceased individuals, while 68.5% of the survivors exhibited a prehospital ROSC. Despite no difference in hospital arrival time, the floor of the building where the arrest occurred was statistically higher for survivors than for non-survivors (6.7 ± 6.0 and 6.2 ± 5.5, respectively). Hospital procedures like primary percutaneous intervention, targeted temperature management, and ECMO (extracorporeal membrane oxygenation) CPR were more common among survivors, whereas non-survivors more frequently received mechanical CPR (Table 1).

**Figure 2:**
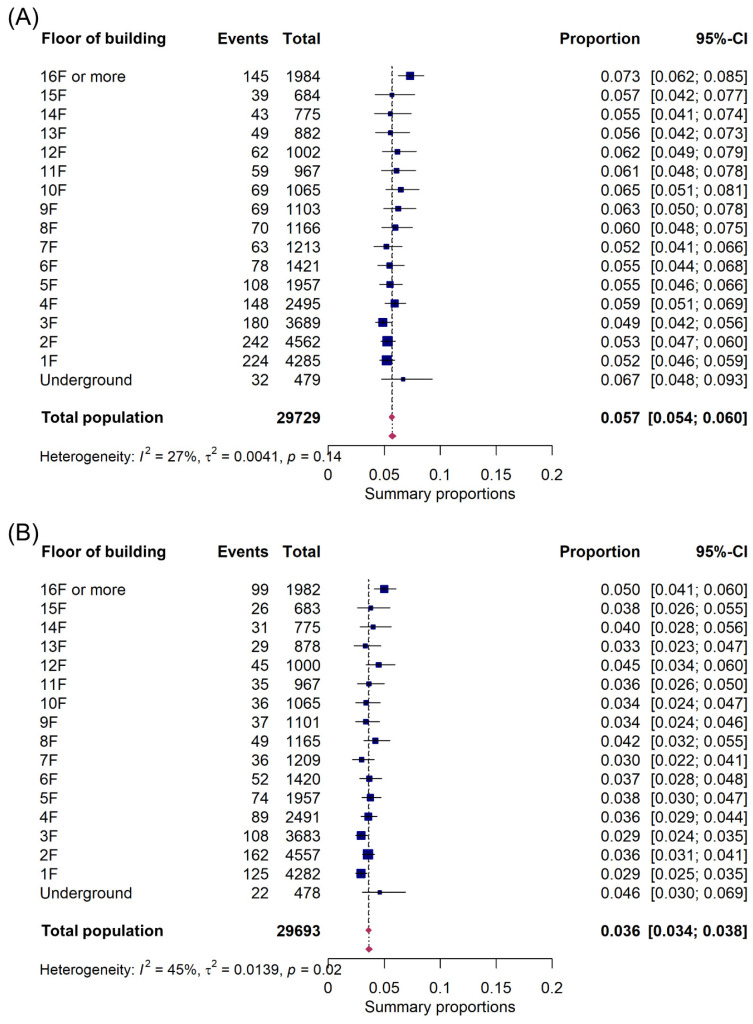
Forest plot of survival to discharge based on building floor of OHCA occurrence. CI, confidence interval. (**A**) Survival to discharge, (**B**) favorable neurological outcome.

Survival to discharge rate by building floor ranged from 4.9% to 7.3%, with an overall survival rate of 5.7%. The survival rate was lowest on the third floor and highest on the 16th floor and above, with the ground floor showing a survival rate of 5.2% (Figure 2A). A subgroup analysis revealed a survival rate of 12.0% among adults younger than 65 years. On the first three floors, survival rates ranged from 9.7% to 10.5%, lower than rates on floors above the third. In contrast, older patients had a survival rate of 2.5%, with no significant difference by floor. Cardiac arrests in residential areas were associated with an overall survival rate of 4.2%, while urban areas had a rate of 5.7% (Supplementary Figure S1).

In terms of favorable neurological outcomes, rates ranged from 2.9% to 5.0%, with an overall rate of 3.6%. The lowest favorable neurological outcomes occurred on the first and third floors, with the highest on the 16th floor and above (Figure 2B). A subgroup analysis showed an 8.9% rate of favorable neurological outcomes among adults younger than 65 years. Older adults had a survival rate of 2.5%, with no significant variation by floor. The overall survival rate for cardiac arrests in residential areas was 2.7%, compared with 3.6% in urban areas (Supplementary Figure S2).

**Figure 3:**
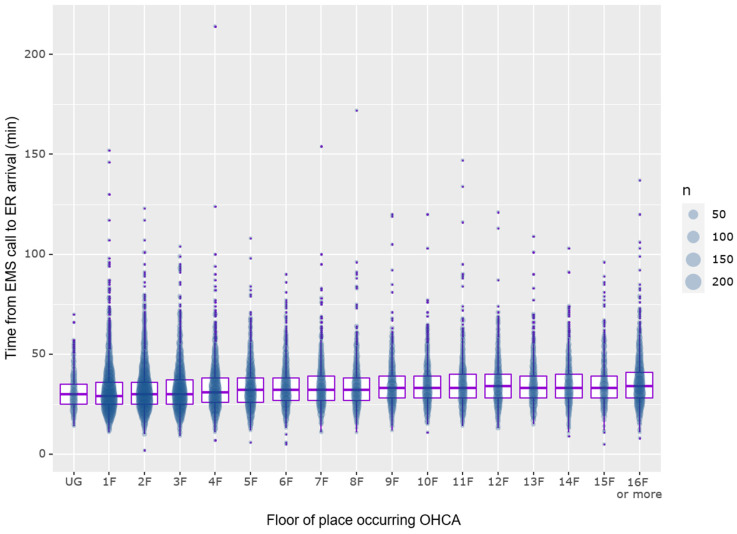
Box plot and population size plot for time interval of hospital arrival time for OHCA patients, sorted by floor of occurrence. EMS, emergency medical services, ER, emergency department, OHCA, out-of-hospital cardiac arrest, UG, underground.

For every one-floor increase, the odds of prehospital ROSC increased by a factor of 1.02 (95% CI 1.01–1.03). However, the effect size suggests that the difference was not dramatic in a practical perspective (Supplementary Table S1). Hospital arrival times increased with each floor, showing a correlation coefficient of 0.124 (*p* < 0.001). Median arrival times were 29.0 (25.0–36.0) minutes on the ground floor and 34.0 (28.0–41.0) minutes on the 16th floor and above (Supplementary Table S2 and Figure 3).

Factors affecting survival to discharge in OHCA events by building floor included age (odds ratio [OR] 0.97, 95% confidence interval [CI] 0.96–0.98, *p* < 0.001), witnessed OHCA (OR 2.33, 95% CI 1.72–3.16, *p* < 0.001), shockable rhythm (OR 6.12, 95% CI 4.61–8.12, *p* < 0.001), prehospital ROSC (OR 53.71, 95% CI 39.84–72.40, *p* < 0.001), and hospital arrival time (OR 0.97, 95% CI 0.95–0.98, *p* < 0.001).

Factors affecting favorable neurological outcome in OHCA events by building floor included age (OR 0.99, 95% CI 0.97–0.99, *p* = 0.035), witnessed OHCA (OR 1.93, 95% CI 1.10–3.38, *p* = 0.022), bystander CPR (OR 1.87, 95% CI 1.02–3.44, *p* = 0.043), shockable rhythm (OR 5.68, 95% CI 3.41–9.46, *p* < 0.001), and prehospital ROSC (OR 5.85, 95% CI 3.62–9.46, *p* < 0.001). These factors were influential after adjusting for other variables (Table 2).

In the subgroups divided into shockable rhythm groups and non-shockable rhythm groups, age, witnessed arrest, and prehospital ROSC were the most important factors that commonly influenced the survival to discharge and favorable neurological outcome (Supplementary Tables S3 and S4).

## 4. Discussion

Our investigation shed light on the nuanced relationship between the building floor level of an OHCA event and survival rates in the context of South Korea’s high-rise residential areas. While our findings demonstrate a weak but statistically significant correlation between the floor level and the time of arrival at the hospital, they suggest the potential impact of the floor level on emergency medical care delivery. Interestingly, despite the longer response times of EMS, patients from higher floors exhibited slightly improved survival rates. This trend may be attributable to younger, healthier residents typically inhabiting higher floors, as supported by the lower average age of survivors and a 12% survival rate among adults under 65 years of age. However, the influence of the floor level was not significant in our multivariate analysis. Instead, age, witnessed OHCA, shockable rhythm, and prehospital ROSC were identified as significant survival predictors. Consequently, our findings call into question the critical role of the building floor in OHCA outcomes. They stress the necessity for targeted improvements in residential emergency response within South Korea, with an emphasis on witness interventions, shockable rhythm management, and the enhancement of prehospital ROSC rates.

Previous research, such as the study by Kobayashi et al., has shown that in buildings with more than three floors, there is an approximately 2 min delay in hospital arrival times relative to buildings with fewer floors [8]. Similarly, Lian et al. demonstrated a longer duration of EMS response in high-rise locations during OHCAs [9]. A Canadian study corroborated these findings by confirming lower survival rates for OHCA patients residing on the third floor or above in comparison to those on lower floors [12]. In a previous study conducted in South Korea [13], study authors investigated the impact of building height on outcomes for OHCA patients. The findings indicated that there was no significant correlation between building height and outcomes in a residential setting. However, in public places, it was observed that lower floors were associated with improved neurological outcome compared to higher floors. Nevertheless, a comprehensive examination of the building’s floor was not conducted, and it was partitioned and contrasted between the levels above and below the third floor. In the present study, our objective was to provide the findings in relation to the increasing variation of floors via a floor-based approach. Our own research found a considerable 5 min discrepancy in EMS response times between the first floor and higher floors in high-rise buildings. This discrepancy is primarily due to time spent on vertical transport, which includes elevator wait times and access periods, rather than the actual elevator travel time. Contrary to expectations, our findings revealed no significant difference in hospital arrival times between OHCA survivors and non-survivors, suggesting that factors such as age and initial cardiac rhythm type may have a more significant impact on survival outcomes. Therefore, it is essential that future research prioritizes strategies to minimize vertical transportation time in multi-story buildings and to investigate other key factors influencing OHCA survival in these settings.

Significant factors impacting survival outcomes after an OHCA were identified in this study, including age, whether a cardiac arrest was witnessed, the presence of a shockable rhythm, prehospital ROSC, and the hospital arrival time [18,19,20,21]. These factors align with findings from prior studies, reinforcing the critical role of immediate bystander intervention, efficient prehospital care, and rapid transport to the hospital in improving OHCA survival outcomes [18,20,22,23,24]. Our research confirmed age as a key predictor of OHCA outcomes, with evidence indicating declining survival rates with advancing age [18,25]. It was found that the building’s number of floors did not significantly affect survival rates among older patients. Interestingly, the third floor was associated with the lowest survival rate among younger adults. Explanations for these results are challenging to determine from a simple mortality rate comparison by floor. However, the lower floors’ data could reflect not only residences but also commercial facilities and factories, potentially influencing the shape of the building, the location of the event, and EMS accessibility, among other factors [26].

The age factor still affects OHCA. As age increases, survival rates were shown to decrease. Especially in the older patients, it did not appear to be significantly affected by the floor level, showing a similar mortality rate regardless of the floor. However, among adults, the lowest survival rate was shown on the third floor. One thing that can be estimated is that, in Korea, the most common type of residence after apartments is terraced houses with fewer floors. Buildings with less than five floors often do not have elevators, which can be a factor that reduces the accessibility of EMS, and this can explain why the second to fourth floors showed relatively lower survival rates compared to other floors. However, time did not show an increased pattern compared to other floors, and we could not find sufficient evidence to support this. If we could clearly distinguish the types of residences and identify the paths of movement within the buildings, a detailed analysis could be conducted.

Our study highlighted the relatively low utilization rate of AEDs. Given that South Korea is a country with a high prevalence of apartment buildings, and the installation of AEDs is mandated for complexes housing over 500 units [27,28], this is a significant finding. The importance of these legal requirements is only growing as more Korean households choose apartment living. Apartment complexes with 500 or more households are mandated to install AEDs, and as per the most recent data, 31.0% are compliant [29]. However, a study by Oh et al. reported that actual AED usage in these buildings was a mere 0.13% [30]. Our own research found the overall AED usage frequency so low that it was challenging to analyze usage by floor. This disparity highlights the urgent need for practical solutions that address the barriers to AED usage. For instance, residents may be unaware of the AED location, or perhaps they lack the training and confidence to use the devices during an emergency. Consequently, it is crucial that efforts are made to enhance AED usage in multi-story buildings, which includes planning optimal floor layouts to maximize AED accessibility and utilization.

The implications of our findings are extensive. They primarily emphasize the need for custom emergency response strategies in high-rise buildings, particularly in urban settings. These strategies may include improved building designs to expedite EMS access, increased availability of AEDs on every floor, and comprehensive community education about immediate bystander intervention. Furthermore, our findings accentuate the potential of leveraging digital technologies, such as smartphone applications, to alert bystanders to a nearby cardiac arrest, thereby significantly improving response times and survival outcomes.

This study faced several limitations. First, while we relied on a comprehensive national dataset, it is important to note that these data were originally generated by healthcare providers in the field. This situation inherently may lead to a higher probability of missing or incomplete data and introduces potential biases in the reporting. Also, the time of arrival at the scene was important because that it may vary depending on the building floor, but the registry did not have information about it. The given time information had limitations in interpreting the results. Second, we encountered limitations in terms of the available information regarding the building structure. For instance, detailed data, including the total number of floors in a building and specifications about internal movement, such as the existence of an elevator, were not available. As a result, we were compelled to depend on incomplete data about the number of floors. Notably, in the case of low-rise buildings (those with fewer than five floors), they often lack elevators, which necessitates emergency transport via stairs. This can impose significant challenges to efficient evacuation and could potentially influence survival rates. This is an aspect that was not fully examined in our research. Thirdly, our study was unable to provide a detailed representation of the individual hospital treatment process. This was largely due to restricted access to these kinds of data, which also rendered us unable to analyze the impact of factors such as medication use and hospital events on the overall treatment outcomes. Our fourth limitation pertains to generalizability. While our study was designed to report on South Korean data characteristics, these findings may not easily extend to other countries’ contexts due to the unique nature of residences and building structures in Korea. Lastly, it is worth mentioning that as this was a retrospective study, there was a possibility of selection bias and the existence of hidden confounding factors. These could potentially distort the findings or limit the overall conclusions of our study.

## 5. Conclusions

Our findings suggest that the floor level where an OHCA occurs can affect survival rates and the time of arrival at the hospital within high-rise living spaces. However, additional research is essential to unravel the precise mechanisms contributing to these observations. These findings underscore the necessity for refining EMS strategies and building safety protocols in high-rise living environments. Moreover, enhancing community awareness about immediate bystander intervention and CPR can potentially improve the survival outcomes of OHCA patients.

## Figures and Tables

**Table 1:** Baseline patient characteristics.

Factors	Death (n = 28,049)	Survival (n = 1680)	*p* Value
Sex, male (%)	16,886 (60.2%)	1264 (75.2%)	<0.001
Age, mean	68.9 ± 18.3	55.4 ± 17.0	<0.001
Urban	27,585 (98.3%)	1668 (99.3%)	0.004
Floor of building occurring arrest	6.2 ± 5.5	6.7 ± 6.0	<0.001
Witnessed cardiac arrest	11,747 (41.9%)	1346 (80.1%)	<0.001
Shockable rhythm	2214 (7.9%)	1086 (64.6%)	<0.001
Dispatch CPR	22,750 (81.1%)	1405 (83.6%)	0.011
Bystander CPR	8004 (28.5%)	914 (54.4%)	<0.001
Bystander AED	20 (0.1%)	6 (0.4%)	0.066
Arrest in public places	448 (1.6%)	61 (3.6%)	
Cause of arrest, cardio-genic	26,156 (93.3%)	1608 (95.7%)	<0.001
Prehospital ROSC	551 (2.0%)	1150 (68.5%)	<0.001
Hospital arrival time (call to hospital arrival)	33.0 ± 10.3	33.1 ± 12.1	0.693
Underlying disease			
Hypertension	10,608 (37.8%)	667 (39.7%)	0.129
Diabetes mellitus	7538 (26.9%)	341 (20.3%)	<0.001
Heart disease	5113 (18.2%)	435 (25.9%)	<0.001
Renal disease	2128 (7.6%)	107 (6.4%)	0.073
Respiratory disease	2265 (8.1%)	96 (5.7%)	0.001
Stroke	2822 (10.1%)	122 (7.3%)	<0.001
Dyslipidemia	1042 (3.7%)	154 (9.2%)	<0.001
In-hospital procedures			
Primary PCI	232 (0.8%)	349 (20.8%)	<0.001
TTM	545 (1.9%)	490 (29.2%)	<0.001
Mechanical CPR	3664 (13.1%)	73 (4.3%)	<0.001
ECMO CPR	210 (0.7%)	54 (3.2%)	<0.001

AED, automated external defibrillator; CPR, cardiopulmonary resuscitation; ECMO, extracorporeal membrane oxygenation; PCI, percutaneous coronary intervention; ROSC, return of spontaneous circulation; TTM, target temperature management.

**Table 2:** Univariate and multivariate logistic regression analysis of survival to discharge and favorable neurological outcome for patients with OHCA.

Factor	Univariate OR (95% CI)	*p* Value	Adjusted OR (95% CI)	*p* Value
Outcome: Survival to discharge				Nagelkerke R^2^ = 0.636
Age	0.97 (0.97–0.97)	<0.001	0.97 (0.96–0.98) *	<0.001
Sex	2.01 (1.79–2.25)	<0.001	1.09 (0.81–1.46)	0.560
Urban	2.34 (1.32–4.16)	0.003	0.47 (0.06–3.97)	0.487
Floor of building	1.02 (1.01–1.02)	<0.001	1.00 (0.98–1.02)	0.910
Witnessed OHCA	5.53 (4.89–6.27)	<0.001	2.33 (1.72–3.16) *	<0.001
Bystander CPR	2.70 (2.25–3.28)	<0.001	1.01 (0.72–1.39)	0.978
Shockable rhythm	20.19 (17.48–23.35)	<0.001	6.12 (4.61–8.12) *	<0.001
Cause of arrest, cardiogenic	1.59 (1.25–2.05)	<0.001	1.40 (0.82–2.40)	0.219
Public place	2.32 (1.75–3.02)	<0.001	0.88 (0.43–1.77)	0.711
Prehospital ROSC	108.29 (94.88–123.80)	<0.001	53.71 (39.84–72.40) *	<0.001
Hospital arrival time (call to hospital arrival)	1.00 (1.00–1.01)	0.648	0.97 (0.95–0.98) *	<0.001
Outcome: Favorable neurological outcome				Nagelkerke R^2^ = 0.449
Age	0.97 (0.97–0.97)	<0.001	0.99 (0.97–0.99) *	0.035
Sex	2.01 (1.80–2.25)	<0.001	1.47 (0.86–2.49)	0.156
Urban	2.34 (1.38–4.40)	0.004	0.15 (0.02–2.82)	0.095
Floor of building	1.02 (1.01–1.02)	<0.001	1.01 (0.98–1.02)	0.896
Witnessed OHCA	5.53 (4.89–6.27)	<0.001	1.93 (1.10–3.38) *	0.022
Bystander CPR	2.70 (2.25–3.28)	<0.001	1.87 (1.02–3.44) *	0.043
Shockable rhythm	20.19 (17.48–23.35)	<0.001	5.68 (3.41–9.46) *	<0.001
Cause of arrest, cardiogenic	1.59 (1.25–2.05)	<0.001	1.92 (0.54–6.77)	0.312
Public place	2.32 (1.75–3.02)	<0.001	1.67 (0.53–5.32)	0.385
Prehospital ROSC	20.19 (17.48–23.35)	<0.001	5.85 (3.62–9.46) *	<0.001
Hospital arrival time (call to hospital arrival)	1.00 (1.00–1.01)	0.648	1.00 (0.98–1.02)	0.81

Adjusted for sex, age, urban, floor of building, witnessed OHCA, bystander CPR, shockable rhythm, cardiogenic cardiac arrest, public place, prehospital ROSC, and the time from EMS call to ER arrival. * Factors included in the final logistic regression model for survival to discharge.

## Data Availability

The datasets generated during the study presented herein are available from the corresponding author upon reasonable request.

## Supplementary Materials

The following supporting information can be downloaded at: https://www.mdpi.com/article/10.3390/jpm13081265/s1. Table S1: Odds ratio of floor level of building for prehospital ROSC, Table S2: Correlation between hospital arrival time and floor level of building, Table S3: Univariate and multivariate logistic regression analysis of survival to discharge and favorable neurological outcome for OHCA patients with shockable rhythm, and Table S4: Univariate and multivariate logistic regression analysis of survival to discharge and favorable neurological outcome for OHCA patients with non-shockable rhythm, Figure S1: Forest plot of subgroup analysis for survival to discharge based on building floor of OHCA occurrence, and Figure S2: Forest plot of subgroup analysis for favorable neurological outcome based on building floor of OHCA occurrence.
