# Supplementary material for: The Influence of Cardiac Arrest Floor-Level Location within a Building on Survival Outcomes
Source: J Pers Med. 2023 Aug 16;13(8):1265. doi: 10.3390/jpm13081265 (PMC10455151; doi:10.3390/jpm13081265)
Supplement: Supplementary file 1 [file jpm-13-01265-s001.zip › jpm-2559395-supplementary.pdf]

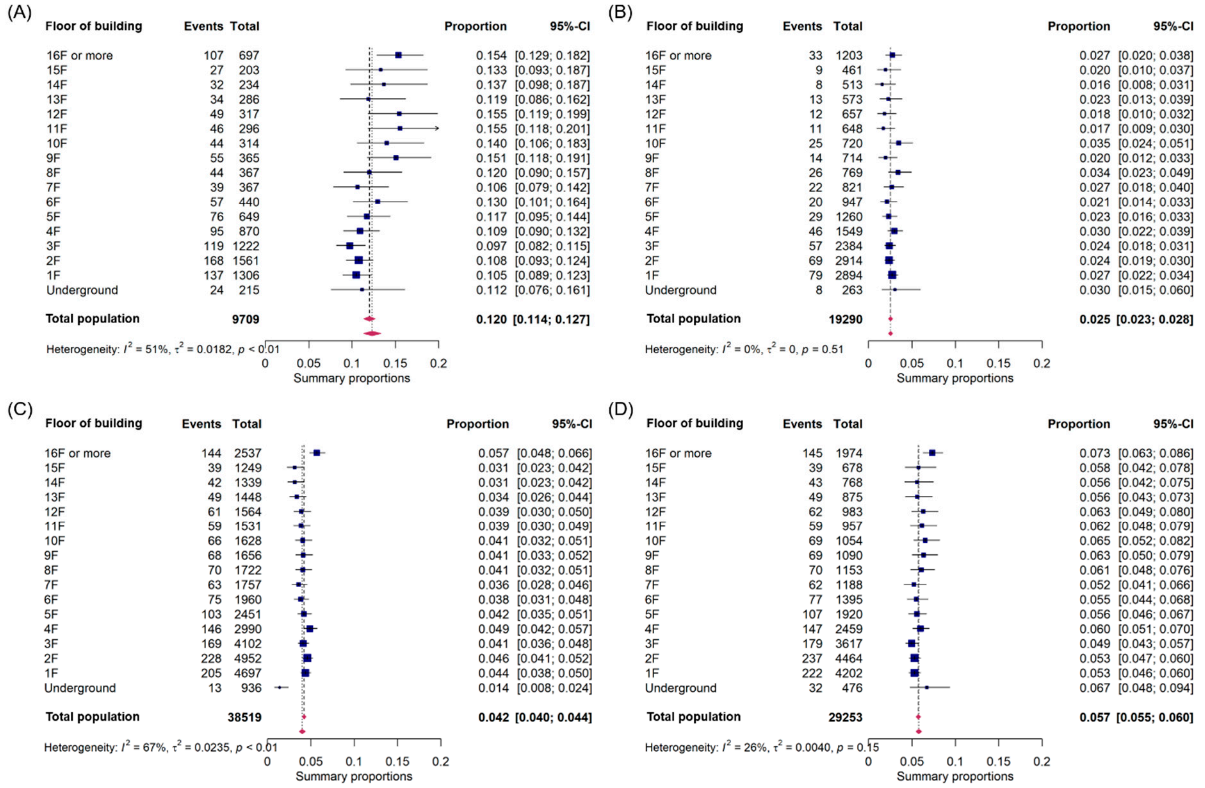

Supplementary Figure S1. Forest plot of subgroup analysis for survival to discharge based on building floor of OHCA occur-rence. Subgroups include (A) adult (ages 18-64), (B) older adults (aged 65 and above), (C) residen-tial area, (D) urban setting. CI, confidence interval.

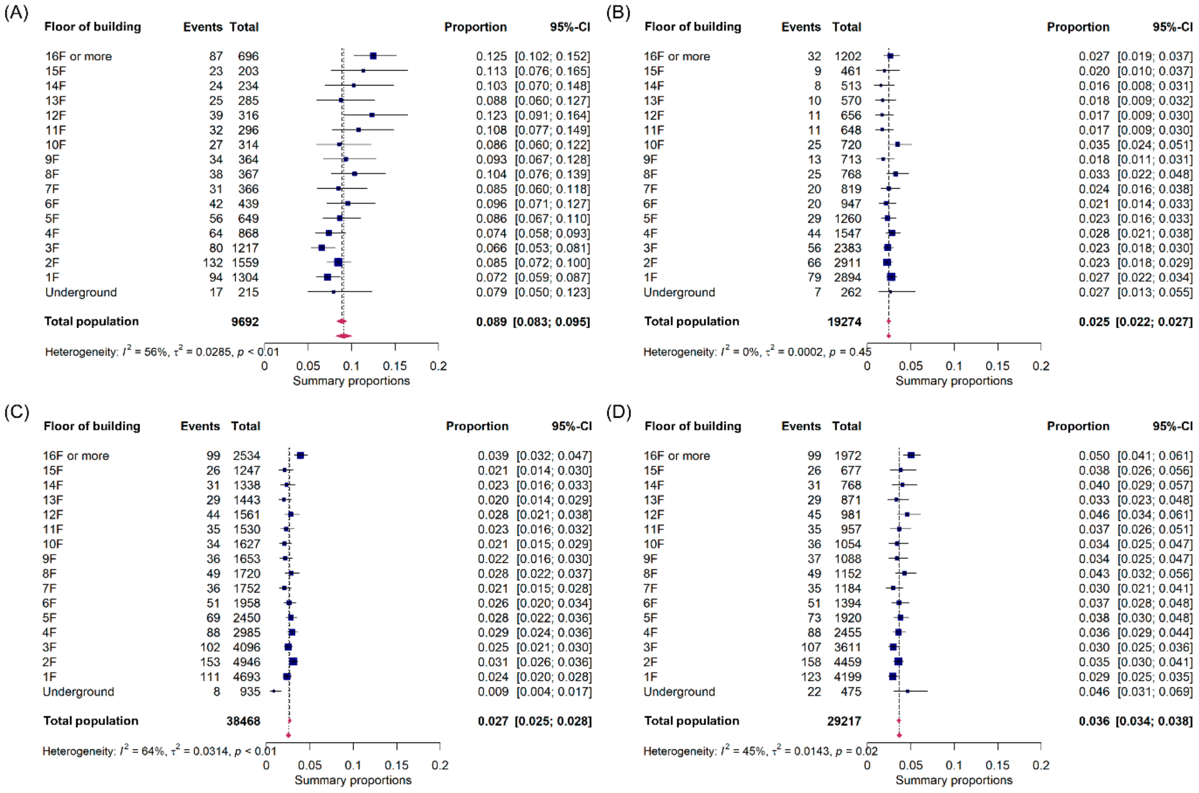

Supplementary Figure S2. Forest plot of subgroup analysis for favorable neurological outcome based on building floor of OHCA occurrence. Subgroups include (A) adult (ages 18-64), (B) older adult (aged 65 and above), (C) residential area, (D) urban setting. CI, confidence interval.

Supplementary Table S1. Odds ratio of floor level of building for prehospital ROSC.

Floor of building	No prehospital ROSC (N=28028)	Prehospital ROSC (N=1701)
16F or more	1842 (92.8)	142 (7.2)
15F	645 (94.3)	39 (5.7)
14F	723 (93.3)	52 (6.7)
13F	835 (94.7)	47 (5.3)
12F	935 (93.3)	67 (6.7)
11F	905 (93.6)	62 (6.4)
10F	1003 (94.2)	62 (5.8)
9F	1030 (93.4)	73 (6.6)
8F	1092 (93.7)	74 (6.3)
7F	1156 (95.3)	57 (4.7)
6F	1341 (94.4)	80 (5.6)
5F	1840 (94.0)	117 (6.0)
4F	2342 (93.9)	153 (6.1)
3F	3497 (94.8)	192 (5.2)
2F	4304 (94.3)	258 (5.7)
1F	4091 (95.5)	194 (4.5)
Underground	447 (93.3)	32 (6.7)
**Crude OR 1.02, 95% CI 1.01-1.03, p<0.001**		

Supplementary Table S2. Correlation between hospital arrival time and floor level of building.

Floor of building	Hospital arrival time*, median [IQR]
16F or more	34.0 [28.0;41.0]
15F	33.0 [28.0;39.0]
14F	33.0 [28.0;40.0]
13F	33.0 [28.0;39.0]
12F	34.0 [28.0;40.0]
11F	33.0 [28.0;40.0]
10F	33.0 [28.0;39.0]
9F	33.0 [28.0;39.0]
8F	32.0 [27.0;38.0]
7F	32.0 [27.0;39.0]
6F	32.0 [27.0;38.0]
5F	32.0 [26.0;38.0]
4F	31.0 [26.0;38.0]
3F	30.0 [25.0;37.0]
2F	30.0 [25.0;36.0]
1F	29.0 [25.0;36.0]
Underground	30.0 [25.0;35.0]
**Pearson's correlation** between the floor of building and the Hospital arrival time	0.124 ($p < 0.001$)

Supplementary Table S3. Univariate and multivariate logistic regression analysis of survival to discharge and favorable neurological outcome for OHCA patients with shockable rhythm.

Factor	Univariate OR (95% CI)	*p* Value	Adjusted OR (95% CI)	*p* Value
**Outcome: Survival to discharge**				
Age	0.95 (0.95-0.96)	<0.001	**0.95 (0.93-0.96)***	<0.001
Sex	1.48 (1.17-1.89)	<0.001	1.27 (0.76-2.15)	0.359
Urban	3.74 (1.03-23.97)	0.083	0.30 (0.03-6.34)	0.325
Floor of building	1.02 (1.00-1.04)	0.011	0.99 (0.95-1.02)	0.424
Witnessed OHCA	3.15 (2.45-4.09)	<0.001	**2.16 (1.28-3.69)***	0.004
Bystander CPR	2.27 (1.60-3.27)	<0.001	0.86 (0.50-1.50)	0.580
Cause of arrest, cardiogenic	4.06 (1.39-17.22)	0.024	**5.27 (0.80-48.08)***	0.106
Public place	0.78 (0.48-1.23)	0.293	-	-
Prehospital ROSC	63.46 (47.25-86.32)	<0.001	**77.21 (49.44-124.66)***	<0.001
Hospital arrival time (call to hospital arrival)	0.98 (0.97-0.99)	<0.001	**0.97 (0.95-0.98)***	<0.001
**Outcome: Favorable neurological outcome**				
Age	0.95 (0.95-0.96)	<0.001	**0.96 (0.95-0.97)***	<0.001
Sex	1.77 (1.35-2.33)	<0.001	**1.74 (1.05-2.90)***	0.032
Urban	2.64 (0.73-16.93)	0.202	-	-
Floor of building	1.03 (1.01-1.04)	0.002	1.00 (0.97-1.03)	0.910
Witnessed OHCA	3.38 (2.55-4.57)	<0.001	**2.36 (1.39-4.06)***	0.002
Bystander CPR	2.35 (1.61-3.51)	<0.001	0.88 (0.50-1.58)	0.672
Cause of arrest, cardiogenic	9.45 (1.98-169.25)	0.028	8774468.98 (0.00-)	0.976
Public place	0.87 (0.52-1.42)	0.597	-	-
Prehospital ROSC	44.73 (33.58-60.30)	<0.001	**43.14 (28.99-65.75)***	<0.001
Hospital arrival time (call to hospital arrival)	0.99 (0.98-0.99)	0.002	0.99 (0.97-1.01)	0.216

Supplementary Table S4. Univariate and multivariate logistic regression analysis of survival to discharge and favorable neurological outcome for OHCA patients with non-shockable rhythm.

Factor	Univariate OR (95% CI)	*p* Value	Adjusted OR (95% CI)	*p* Value
**Outcome: Survival to discharge**				
Age	0.98 (0.98-0.99)	<0.001	**0.98 (0.97-0.99)***	<0.001
Sex	1.18 (0.94-1.48)	0.158	1.00 (0.70-1.44)	0.560
Urban	446906.72 (270.81-)	0.955	-	-
Floor of building	1.00 (0.98-1.02)	0.825	1.00 (0.98-1.02)	0.910
Witnessed OHCA	3.17 (2.50-4.05)	<0.001	**2.47 (1.70-3.62)***	<0.001
Bystander CPR	1.46 (1.02-2.13)	0.042	1.07 (0.72-1.62)	0.755
Cause of arrest, cardiogenic	0.68 (0.47-1.01)	0.024	1.01 (0.59-1.80)	0.958
Public place	1.59 (0.71-3.05)	0.204	-	-
Prehospital ROSC	45.66 (35.16-59.33)	<0.001	**44.19 (28.85-68.20)***	<0.001
Hospital arrival time (call to hospital arrival)	0.99 (0.98-1.00)	0.046	**0.96 (0.94-0.98)***	<0.001
**Outcome: Favorable neurological outcome**				
Age	0.98 (0.97-0.98)	<0.001	**0.97 (0.96-0.99)***	<0.001
Sex	1.31 (0.87-2.01)	<0.001	-	-
Urban	359678.36 (0.59-)	0.973	-	-
Floor of building	0.99 (0.95-1.03)	0.675	-	-
Witnessed OHCA	6.27 (3.81-10.95)	<0.001	**2.69 (1.18-6.67)***	0.024
Bystander CPR	8.57 (2.62-52.73)	<0.001	**6.75 (1.92-43.07)***	0.011
Cause of arrest, cardiogenic	1.34 (0.60-3.81)	0.524	-	-
Public place	1.30 (0.21-4.15)	0.714	-	-
Prehospital ROSC	189.66 (115.66-326.56)	<0.001	**170.01 (73.74-463.79)***	<0.001
Hospital arrival time (call to hospital arrival)	0.99 (0.97-1.01)	0.457	-	-
